# Using Tensor Completion Method to Achieving Better Coverage of Traffic State Estimation from Sparse Floating Car Data

**DOI:** 10.1371/journal.pone.0157420

**Published:** 2016-07-22

**Authors:** Bin Ran, Li Song, Jian Zhang, Yang Cheng, Huachun Tan

**Affiliations:** 1 Jiangsu Key Laboratory of Urban ITS, Jiangsu Province Collaborative Innovation Center of Modern Urban Traffic Technologies, Jiangsu Province Collaborative Innovation Center for Technology and Application of Internet of Things, Room 213, Transportation Building, Southeast University, No. 2 Si Pai Lou, Nanjing, 210096, P.R. China; 2 Department of Transportation Engineering, Beijing Institute of Technology, Beijing, 100081, P.R. China; Chongqing University, CHINA

## Abstract

Traffic state estimation from the floating car system is a challenging problem. The low penetration rate and random distribution make available floating car samples usually cover part space and time points of the road networks. To obtain a wide range of traffic state from the floating car system, many methods have been proposed to estimate the traffic state for the uncovered links. However, these methods cannot provide traffic state of the entire road networks. In this paper, the traffic state estimation is transformed to solve a missing data imputation problem, and the tensor completion framework is proposed to estimate missing traffic state. A tensor is constructed to model traffic state in which observed entries are directly derived from floating car system and unobserved traffic states are modeled as missing entries of constructed tensor. The constructed traffic state tensor can represent spatial and temporal correlations of traffic data and encode the multi-way properties of traffic state. The advantage of the proposed approach is that it can fully mine and utilize the multi-dimensional inherent correlations of traffic state. We tested the proposed approach on a well calibrated simulation network. Experimental results demonstrated that the proposed approach yield reliable traffic state estimation from very sparse floating car data, particularly when dealing with the floating car penetration rate is below 1%.

## 1 Introduction

Real-time traffic state is pivotal in many Intelligent Transportation Systems (ITS) [1] applications: incident detection, vehicle navigation, driver assistance systems [2], and so on. In practice, traffic state can be estimated from fixed traffic detectors and mobile traffic detectors [3]. A fixed detector such as a loop detector has a limited coverage, and it is costly in installation and maintenance. With the development of V2V/V2I and connected vehicle [4], a mobile traffic detector such as the floating car can provide high quality information with a relatively low cost for real-time traffic management and monitoring. Thus the floating car is increasingly applied to traffic data collection and traffic state estimation.

To acquire the entire network traffic state, a sufficient number of floating cars are needed for each link at each time interval. But in practice, floating cars cannot cover the entire road network in a sampling interval due to its limited penetration rate and erratic driving [5–6]. Consequently, floating car data (FCD) is often sparse since many regions and time periods are not covered by probe vehicles and hence the complete traffic state of the road network cannot be obtained by only utilizing the floating car system.

Given the FCD sparsity, researchers proposed some methods to improve the coverage of the traffic state estimation from incomplete FCD based on the correlations of traffic data. Many previous works found that traffic volumes and traffic states have strong correlations in temporal dimensions and spatial dimensions (e.g. link mode, day mode, week mode) [3, 6, 7–15]. And mining these multi-mode similarities of traffic state will make great contributions to uncovered regions’ traffic state estimation. Xing et al. [14] proposed two repair algorithms, named Time-Iteration repair algorithm and Topology-Detecting repair algorithm, utilizing temporal correlations and spatial correlations of FCD respectively, which improved the network travel time coverage from 70% to 90%. Lv et al. [3,6] developed a matching model based on history FCD to compensate the incomplete data, it promoted network speed coverage in different daytime. Qiu et al. [7, 13] proposed a speed fusion method based on principle component analysis (PCA) and neural network (NN) to fuse the speeds of correlated road sections to get the speed of uncovered regions. In addition, Qu et al. [16, 17] proposed two algorithms named BPCA (Bayesian Principal Component Analysis) and PPCA (Probabilistic Principal Component Analysis) to estimate incomplete traffic volume data, they make use of spatial-temporal correlation from matrix volume model. Although these methods improve the coverage of traffic state estimation to some extent, they are still unable to obtain the traffic state of the entire road network since the spatial-temporal correlations of the traffic data is not fully utilized.

Recently, tensor completion methods, which are capable of fully representing the multi-dimension correlations in various datasets, have attracted a lot of interests [8, 18–21], and have been applied to image completion [18, 19], video completion [20], and traffic data completion [8, 21, 22], etc. Tensor is also named multi-way matrix. It can combine and utilize multi-mode correlations of data through preserving the multi-way properties of data and extracting the underlying factors in each mode. Many researchers have developed a series of tensor-based missing data completion methods. Liu et al. [18] proposed a low rank tensor completion algorithm (LRTC) to estimate the unobserved entries of a tensor. Acar et al. [20] proposed an algorithm called Weighted CP Decomposition (CP-WOPT) utilizing CP decomposition and first-order optimization to address the missing data problem. And some researchers developed tensor completion algorithms in estimating missing traffic data. Tan et al. [8] proposed a Tucker decomposition based imputation method (TDI) to complete the missing values of the traffic volume data and the results showed its superior performance over matrix-based methods.

In this paper, we formulate the traffic state estimation as a missing data imputation problem. A tensor model is constructed where observed entries are calculated by the data collected by floating car system and missing entries indicated the uncovered space-time points. The constructed traffic state tensor not only keeps the original structure of the traffic state but also fully encodes the multi-mode correlations of traffic state. Once the tensor model has been built, a typical tensor completion algorithm named Tucker decomposition based imputation method (TDI) [8] is applied to impute the traffic state of the uncovered regions. The TDI method captures the global structure of the data via Tucker decomposition, which is also called high-order singular value decomposition. The precision and efficiency of the proposed method are evaluated on a well calibrated simulation network. Experimental results demonstrated the proposed approach can reliably estimate the missing traffic state from sparse floating car data with a comparable running speed even when the floating car penetration rate decreases to 1%.

This paper is organized as follows. Section 2 presents the methodology of estimating the traffic state. Section 3 discusses the settings of simulation and FCD processing. Section 4 analyzes the traffic state correlations and describes results of experiments. Section 5 concludes this paper.

## 2 Methodology

A framework combining the floating car system with the tensor completion method is proposed to obtain the entire road network traffic state. The floating car system can directly obtain the traffic state of a link at a time interval if there are floating cars passing through it at this time interval. The traffic state of the links that have no floating car passes at a time interval are labeled as missing data. Then the problem of estimating traffic state is cast as completing missing traffic state. Specifically, the proposed approach includes the following three steps:

1. **Constructing a tensor model:** According to the spatial structure of the road network and temporal modes of the traffic state, tensor patterns are introduced to model the traffic state of the entire road network, which can reflect its native structure and encode its multi-mode information in a unified framework.
2. **Labeling missing entries and observed entries of the traffic state tensor:** Some entries of the constructed traffic state tensor are missing due to the sparsity of FCD. At every sampling interval, the entries corresponding to the links through which floating cars pass are labeled as observed entries, and the others are labeled as missing entries. And the value of an observed entry is set as the average speed of all floating cars that pass through the link at a time interval.
3. **Estimating the missing traffic state:** To obtain the traffic state of the entire road network, the missing entries of the traffic state tensor need to be estimated. In this paper, a tensor completion algorithm named TDI is employed for its efficiency of tensor completion in mining the underlying multi-correlations of the traffic state tensor.

The details of the estimating traffic state are given in what follows.

### 2.1 Constructing tensor model

In our study, a road network is represented by a directed graph *G* = {*l*_1_, *l*_2_, …, *l*_*n*_}, *l*_*i*_ represents a pathway called link which is the basic unit of the road networks. A link has attributions such as id, length, and direction and can be described as follows,

L={l|l=l(id,length,direction)}, (1)

where *id* is the unique number which represents the link in the road network, *length* and *direction* stand for the length and direction of the link. The direction takes binary values, 1 or -1.

**Fig 1:**
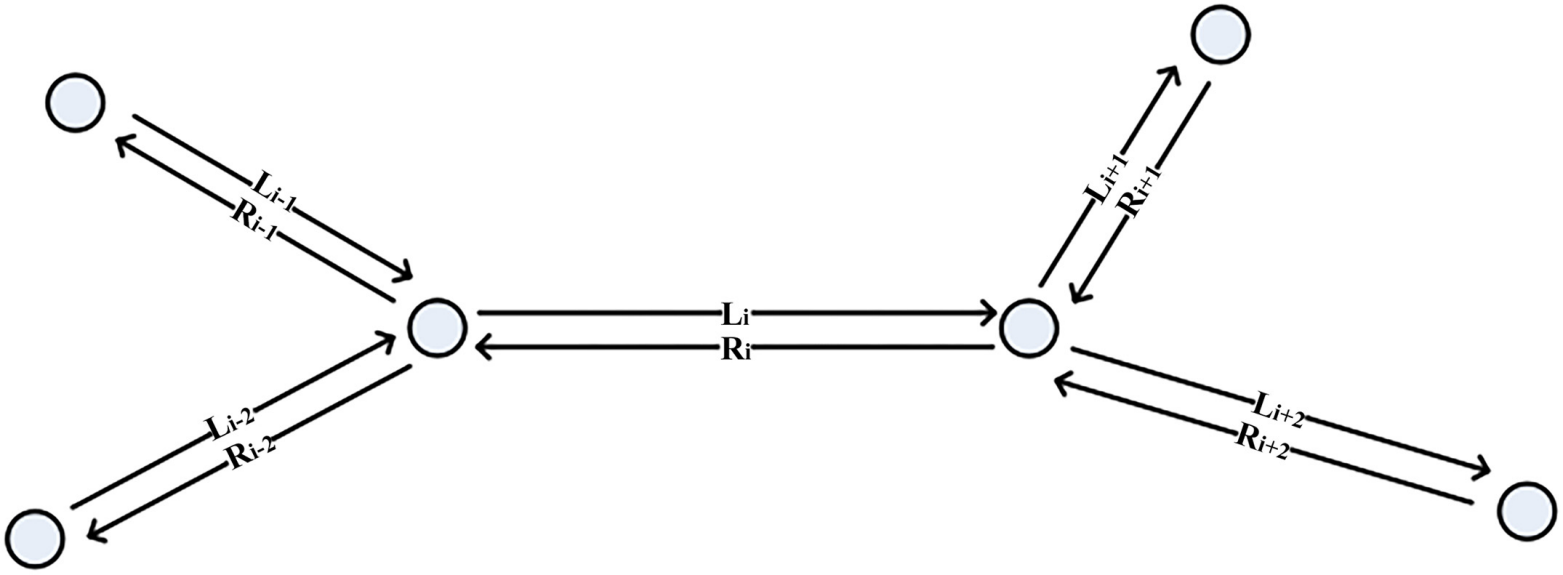
A part of road network.

Fig 1 shows parts of a road network. A circle represents a node, and the arrow indicates the direction of a link. Several links can be connected with each other by one node.

**Fig 2:**
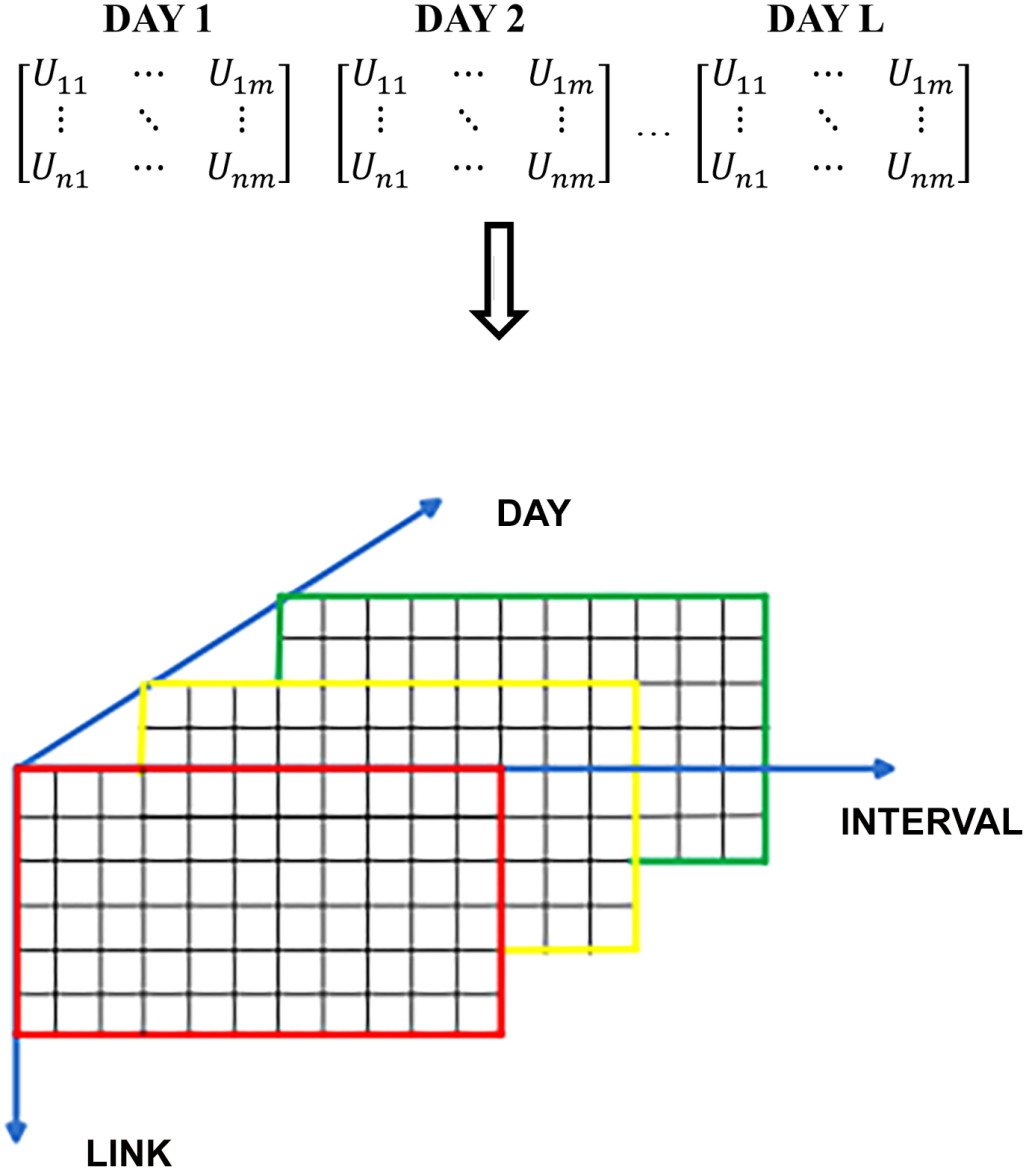
The three-dimension tensor model. L is the number of days.

According to the form of *G*, traffic speed in a day can be written as,

$$\left[\begin{array}{ccc} U_{11} & \cdots & U_{1m}\\ \vdots & \ddots & \vdots \\ U_{n1} & \cdots & U_{nm} \end{array}\right],$$ (2)

where *n* represents the number of links, *m* represents the number of sampling intervals, and *U*_*ij*_ is the traffic speed at interval *j* of link *i*. Put together such matrices in day order, and a tensor model can be established. Fig 2 depicts a 3-D tensor model of traffic state.

Based on the above description, the traffic state tensor can be represented as,

$$A\in {\mathbb{R}}^{\text{DAY}\times \text{LINK}\times \text{INTERVAL}},$$ (3)

A is a three-dimension tensor that has three modes: day mode, link mode, and interval mode. Obviously, this tensor model encodes richer traffic spatial-temporal information than matrix models.

### 2.2 Labeling missing entries and portion area traffic state acquirement

For the traffic state tensor, a lot of entries are missing because there are many links through which no floating cars pass at an interval.

For every sample interval, these links are labeled as missing entries, and the other links are labeled as observed entries. The values of observed entries of tensor are directly calculated from collected floating car data.

Generally, a floating car periodically transmits the real-time GPS information (location, direction, instant speed and time) to the transportation management center. Considering the representation of the road network introduced above, we describe the speed of a floating car in a sending point as

$$V=\{\nu |\nu =\nu \left(l_{i},t_{j},vid\right)\},$$ (4)

where *vid* is the vehicle ID of a floating car, *t*_*j*_ is the element of the set of sampling intervals denoted by *T* = {*t*_1_, *t*_2_, …, *t*_*m*_}, with *t*_*j*_ represented as: $\left(t_{\theta_{j-1}}-t_{\theta_{j}}\right)$, *θ*_*j*-1_ and *θ*_*j*_ is the starting point and ending point of a sampling interval t_j_ respectively, *l*_*i*_ is the link on which floating cars travel in the sampling interval.

In practice, the data from floating cars are clustered spatially according to a GIS digital map which embodies the topological relationship of the urban network. And the link speed is generally calculated by vehicle tracking algorithm [23–25]. In our study, we utilize the mean speed of floating cars as the link speed to simplify the vehicle tracking algorithm, i.e., the link speed (the value of observed entries of the traffic state tensor) of link *l*_*i*_ at time interval *t*_*j*_ can be expressed as

$${\text{U}}_{ij}=\{\begin{array}{rr} \frac{\sum_{z=1}^{\text{num}}\nu \left(l_{i},t_{j},id_{z}\right)}{\text{num}}, & \text{if}\;\;\text{num}\neq 0\\ 0, & \text{if}\;\;\text{num}=0 \end{array}$$ (5)

where num is the number of floating cars travelling on link *l*_*i*_ in time interval *t*_*j*_. In particular, num = 0 means that there is no floating cars passing through a link during this time period. And the corresponding missing entries are assigned to 0 in the tensor model.

### 2.3 Unobserved traffic state estimation

To estimate the missing values in the traffic state tensor, we propose to use a tensor completion algorithm named Tucker decomposition based imputation method (TDI) [8]. TDI algorithm is a first-order weighted optimization algorithm based on tensor decomposition. Tucker decomposition is first introduced by Tucker in 1963 (Tucker, 1963), and then other Tucker decomposition forms have been put forward, in which the principal components in each mode of observed data entries are determined through weighted optimization (WOPT) algorithm of parameters in a latent variable model closely related to gradient analysis [14]. Introduction of WOPT enables Tucker decomposition to analyze the incomplete traffic dataset. By this means, the missing traffic data can be imputed through gradient analysis [8].

For a special three-order tensor, the imputation model based on Tucker decomposition for missing traffic data can be formulated as:

$$ℱ\left(S,X,Y,Z\right)\equiv \text{argmin}\frac{1}{2}∥W*\left(A-S_{\times 1}X_{\times 2}Y_{\times 3}Z\right){∥}_{F}^{2},$$ (6)

where A stands for a three-order tensor of size I_1_ × I_2_ × I_3_ that has missing points. W is an indicating tensor of the same size with A, and it is defined as

$$W_{i_{1}i_{2}i_{3}}=\{\begin{array}{ll} 1 & if\;a_{i_{1}i_{2}i_{3}}is\;known\\ 0 & if\;a_{i_{1}i_{2}i_{3}}is\;missing \end{array}$$ (7)

*i*_1_ = 1, …, *I*_1_, *i*_2_ = 1, …, *I*_2_, *i*_3_ = 1, …, *I*_3_.

In Eq (6), $X\in R^{I_{1}\times R_{1}}$, $Y\in R^{I_{2}\times R_{2}}$, $Z\in R^{I_{3}\times R_{3}}$ are factor matrices and can be thought of as the principal components in each mode,$\;S\in R^{R_{1}\times R_{2}\times R_{3}}$ is the core tensor carrying the level of interaction between different components, and F is the Frobenius norm. The goal of this algorithm is to find factor matrices *X*, *Y*, *Z* and the core tensor S to minimize the objective function in Eq (6). The pseudo-code of TDI based traffic state estimation is given in Algorithm 1, in which A is the original traffic state tensor of missing entries, and $\hat{A}$ is the estimated resulting tensor of complete traffic state. For details about TDI algorithm, please refer to [7].

### Algorithm 1 Tucker Decomposition based Imputation Algorithm (TDI)

**Input**: spares observed traffic state tensor and its missing value indication tensor A, W, multi-linear rank (R_1_,R_2_,R_3_), tolerance *ϵ*_*tol*_, maximum iteration count *k*_*max*_

**Output**: estimated traffic state tensor $\hat{A}$

 1. Initialize the matrices (*X*_0_, *Y*_0_, *Z*_0_) ∈ M. Typically, a good choice of (*X*_0_, *Y*_0_, *Z*_0_) is obtained from the truncated HOSVD.

 2. **For** *k* = 0 to *k*_*max*_ **do**:

 3. Compute $\gamma \;=\;{\left\|B\right\|}^{2},\;with\;B\;=\;W*A$

 4. Compute $C\;=\;W*(S_{\times 1}X_{\times 2}Y_{\times 3}Z)$

 5. **do**

 6. $f\;=\;0.5\gamma -\langle B,C\rangle +0.5{\left\|C\right\|}^{2}$

 7. *w*_*k*_ = *grad F*(*x*_*k*_)

 8. If ${\left\|B-C\right\|}_{W}/{\left\|B\right\|}_{W}<\varepsilon_{tol}$, **then break**

 9. **End for**

 10. Compute the estimated tensor $\hat{A}\;=\;S_{k_{\times 1}}X_{k_{\times 2}}Y_{k_{\times 3}}Z_{k}$

## 3 Data Processing

### 3.1 Simulation test site

**Fig 3:**
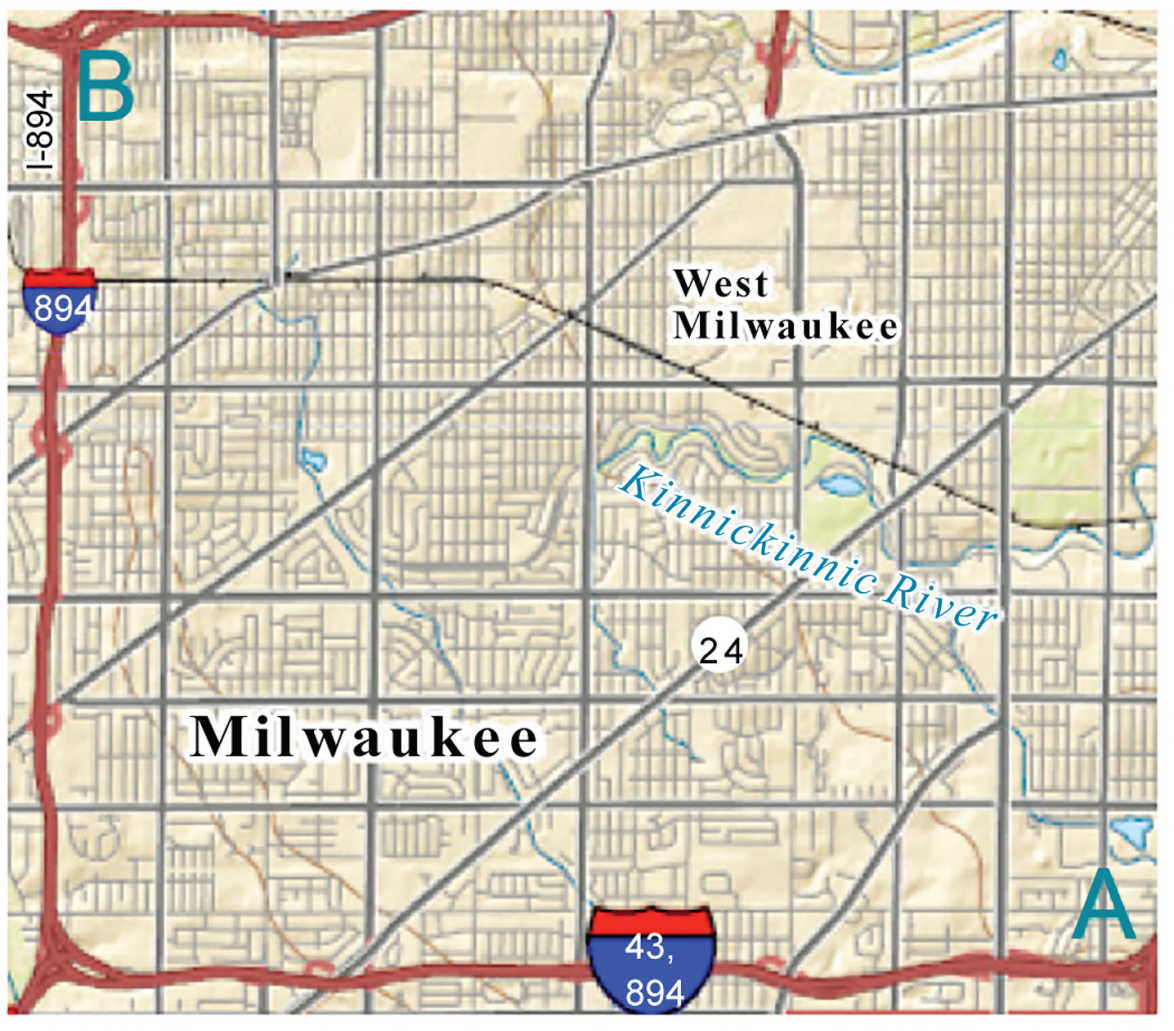
The simulated test site.

A segment of Interstate Highway I-894 near Milwaukee, Wisconsin as shown in Fig 3 (using USGS National Map Viewer) was selected as the test site. This corridor is about 7 miles long, with ramps connecting 12 cross roads. I-894 generally passes through residential areas with the exception of a few commercial districts right next to the freeway. Recurrent congestions occur on several sections of this corridor during peak hours, due to the high traffic volumes.

A replicate corridor was built using the microscopic simulation software VISSIM and calibrated using actual loop detector data from the mainline, entrance and exit ramps. The simulation network was later used to generate the probe vehicle data used in this study.

### 3.2 FCD processing

In the simulation, the network is consisted by 54 links, of which 28 links are in direction 1 (from A to B in Fig 3) and another 26 links are in direction 2 (from B to A in Fig 3). The vehicle data derived from simulation were recorded every 5 seconds. The simulation time covers twelve days, each of which lasts for three hours.

The derived information of all vehicles is used to obtain the true traffic state of road network. The sampling interval is set as one-minute. During each one-minute period, the average speed calculated from all vehicles that travelled through one link is set as the true traffic state of the link, which will be used as the ground truth to evaluate the tensor completion result.

**Fig 4:**
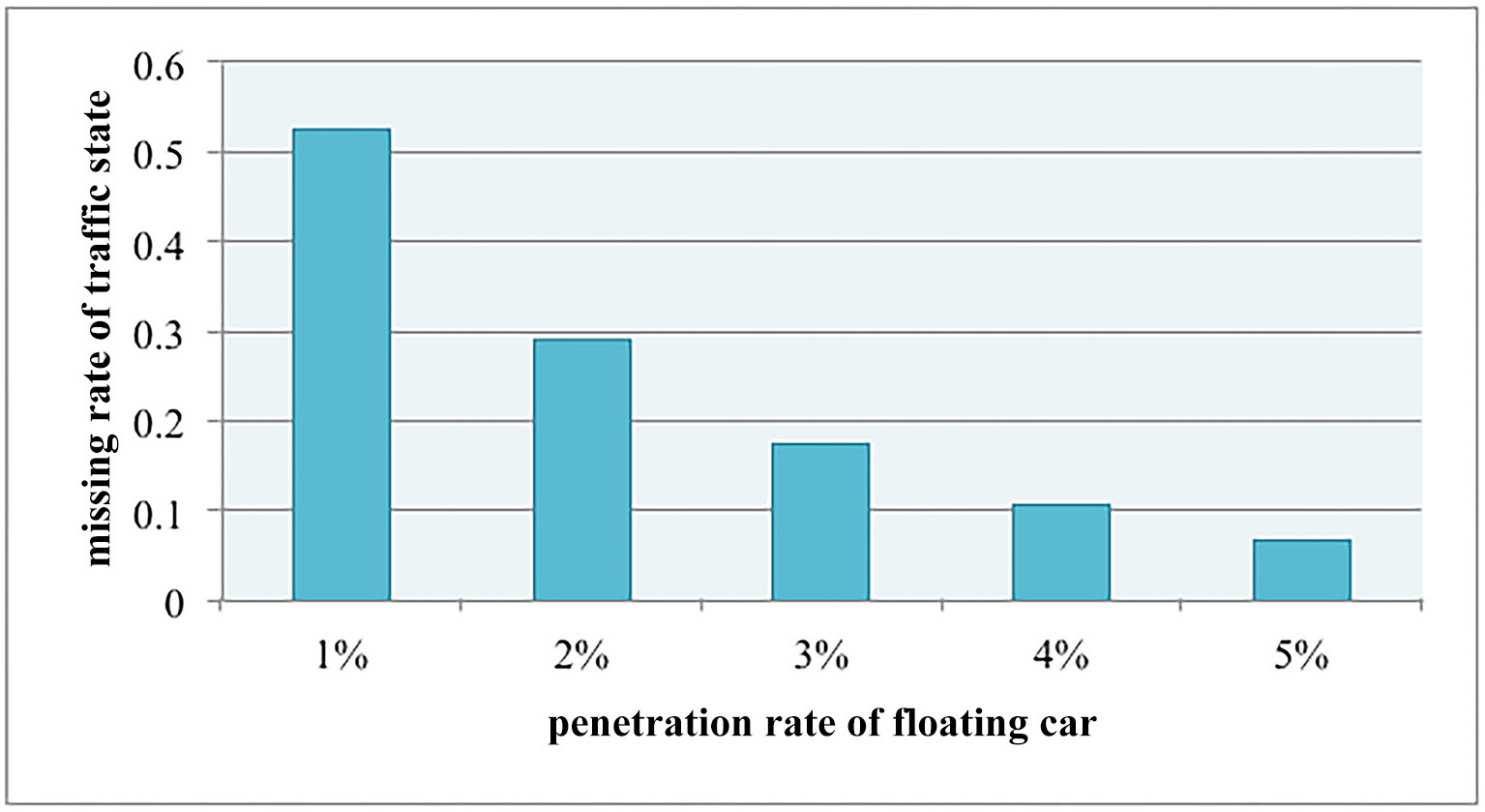
The missing rate in different penetration rate.

However, in practice, the penetration rate of the probe car is often below 10%. Therefore, the traffic state derived by the floating car system could not cover all the links of the road network. For the simulation of the floating car system, five different penetration rates of floating car in the total vehicles are set in our simulation: **1%, 2%, 3%, 4% and 5%.** Then, the states of link with float cars are calculated by the average speed of all floating cars that travelled on the corresponding link in a sampling interval, and the state of link without floating car are set as 0 to indicate missing. Fig 4 gives the processed results of different penetration rates and the corresponding missing data ratio. It shows that the missing rate exceeds 50% when floating car penetration rate is 1%, and when penetration rate equals to 5%, the missing rate is close to 0.1, which is still a quite high ratio. Thus the sparsity of floating car requires to be addressed carefully.

## 4 Experiment

In this section, the traffic state tensor is modeled and the multi-mode correlations are analyzed on the simulated traffic state data to show the low rank characteristics of traffic state tensor. Then some experiments are conducted on the simulated traffic state data to evaluate performances of the proposed method.

### 4.1 Traffic state correlation analysis and traffic state tensor modeling

Several previous researches find that traffic state has strong temporal and spatial regularities of distribution [4, 7, 18, 20, 21, 23]. For example, the link of the same day in different weeks usually experiences similar traffic state, adjacent links also have similar traffic state in the same time. In this subsection, a series of correlation analysis are implemented to investigate the characteristics of traffic state, which include the temporal correlation analysis among different days in one link and the spatial correlation analysis among different links in one day. The correlation coefficient which is formulated in Eq (8) is used to measure the correlations of traffic state,

$$\sigma_{X,Y}=\frac{COV\left(X,Y\right)}{\sigma_{X}\sigma_{Y}}=\frac{E\left[\left(X-E\left(X\right)\right)\left(Y-E\left(Y\right)\right)\right]}{\sqrt{E\left(X^{2}\right)-E^{2}\left(X\right)}\sqrt{E\left(Y^{2}\right)-E^{2}\left(Y\right)}},$$ (8)

From the view of probability and/or statistics, correlation coefficient is a measurement of the strength and direction of the linear relationship between two variables. The larger absolute value of correlation coefficient means the degree of correlation is higher.

**Fig 5:**
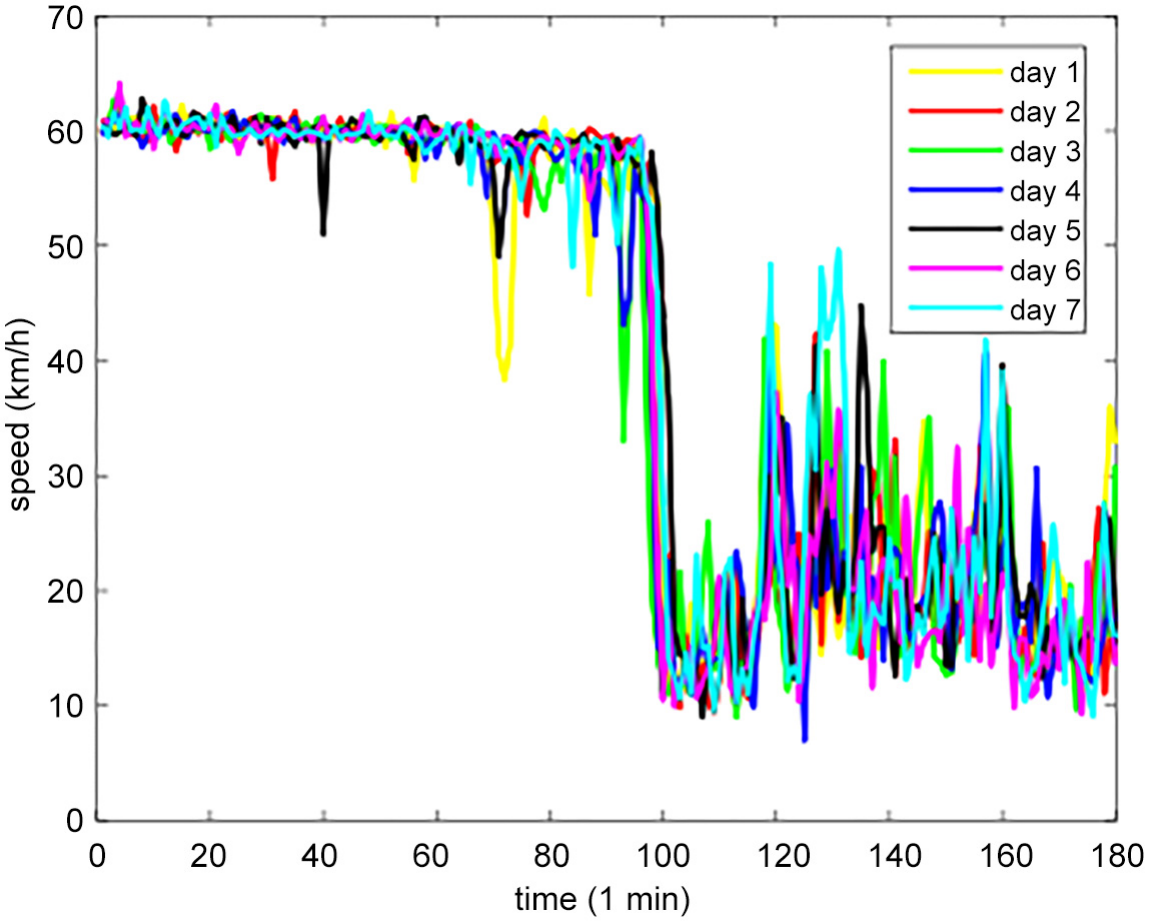
The traffic state trend of a link in different seven days.

In the first experiment, the temporal correlation of traffic state was analyzed: selecting a link randomly and calculating the correlation coefficient between continuous 7 days of the link. The correlation coefficient matrix which represents the degree of correlation between different days is shown in Table 1. The trends of traffic state in the day-mode are illustrated in Fig 5.

**Table 1:** Correlation coefficient matrix of a link in different days.

**DAY**	**1**	**2**	**3**	**4**	**5**	**6**	**7**
**1**	1	0.95	0.93	0.94	0.95	0.95	0.94
**2**	0.95	1	0.96	0.97	0.95	0.97	0.94
**3**	0.93	0.96	1	0.95	0.92	0.95	0.93
**4**	0.94	0.97	0.95	1	0.95	0.96	0.95
**5**	0.95	0.95	0.92	0.95	1	0.96	0.94
**6**	0.95	0.97	0.95	0.96	0.96	1	0.96
**7**	0.94	0.94	0.93	0.95	0.94	0.96	1

**Fig 6:**
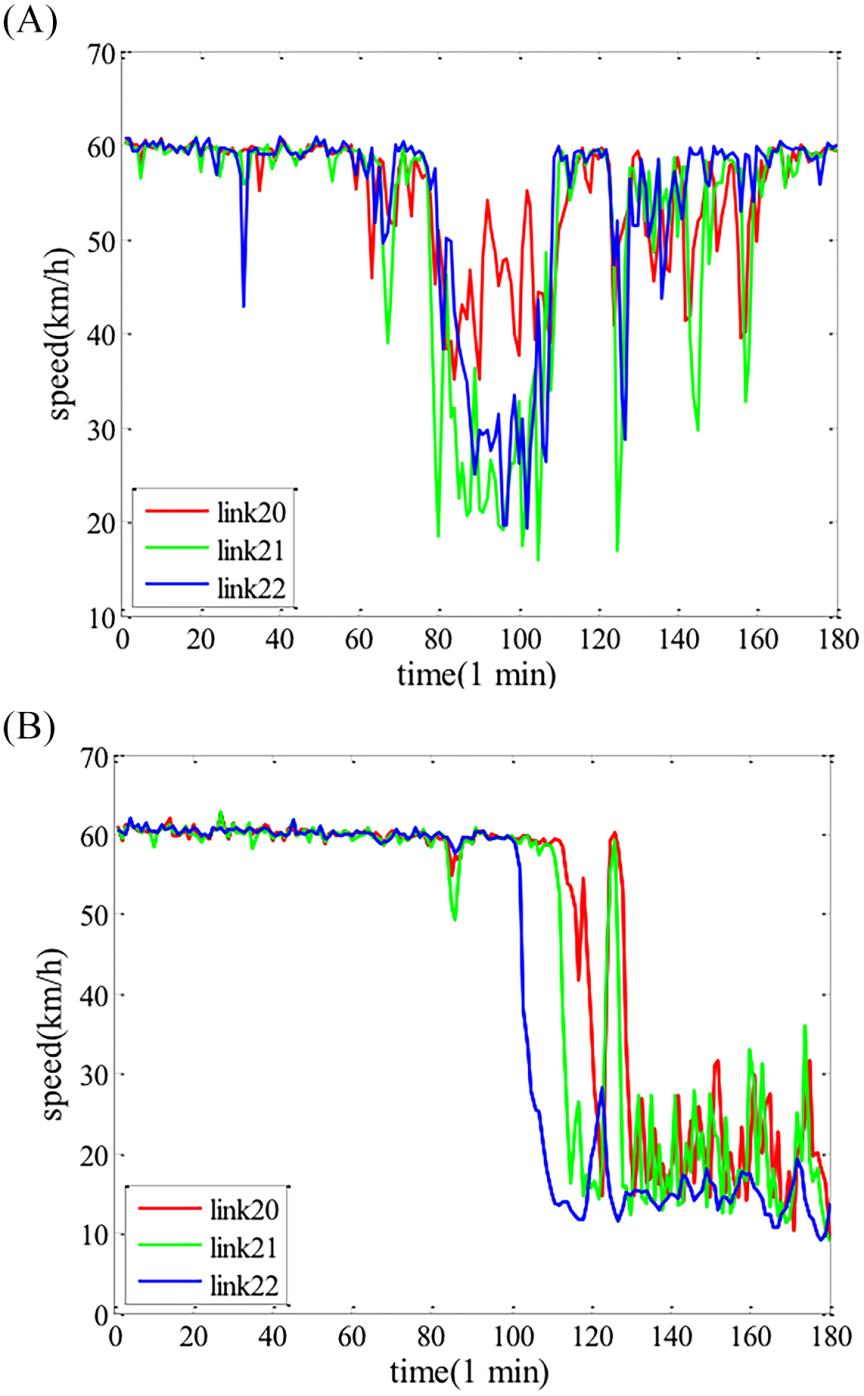
The traffic state trend of adjacent links. a The traffic state trend of three adjacent links in one day. b The traffic state trend of another three adjacent links in one day

In the second experiment, the spatial correlation is investigated. Several adjacent links in a day are randomly selected, and calculated the correlation coefficient between these links in the day. The results are displayed in Table 2, and Fig 6 describes the trends of traffic state in the link-mode.

**Table 2:** Correlation coefficient matrix of adjacent links in one day.

**DAY7**	**Link5**	**Link6**	**Link7**	**DAY1**	**Link20**	**Link21**	**Link22**
**Link5**	1	0.72	0.62	**Link20**	1	0.92	0.81
**Link6**	0.72	1	0.80	**Link21**	0.92	1	0.80
**Link7**	0.62	0.80	1	**Link22**	0.81	0.80	1

From Tables 1 and 2, and Figs 5 and 6, it can be found that traffic state have strong temporal and spatial correlation. Employing tensor form to model the traffic state can keep the original structure of traffic state and fully encode the multi-mode correlation of traffic data.

Our study considered correlation of link mode, day mode and interval mode of traffic state, established a three-dimensional traffic state tensor model:

$$A\in {\mathbb{R}}^{12\times 54\times 180},$$ (9)

where 12 is the number of day, 54 is the number of link and 180 is the total sampling interval of one day.

### 4.2 Experimental setup and result analysis

#### 4.2.1 Experimental Setup

The proposed method has been implemented in Matlab, and all tests were performed on an Intel Core 2 Duo and 2GB RAM computer. Experiments on simulated traffic state data described in Section 3 are conducted to evaluate the performances of proposed method. Two existing methods, Bayesian Principal Component Analysis (BPCA) [17] and Probabilistic Principal Component Analysis (PPCA) [16], are compared with the proposed method.

The performance is evaluated with the root mean squared error (RMSE) and mean absolute percentage error (MAPE) between the estimated data *t*_*est*_ and the true data *t*_*true*_. RMSE and MAPE are defined as:

$$\text{RMSE}=\sqrt{\frac{1}{M}\sum_{m=1}^{M}{\left(t_{true}^{\left(m\right)}-t_{est}^{\left(m\right)}\right)}^{2}}$$ (10)

$$\text{MAPE}=\frac{1}{M}\sum_{m=1}^{M}\left|\frac{t_{true}^{\left(m\right)}-t_{est}^{\left(m\right)}}{t_{true}^{\left(m\right)}}\right|$$ (11)

where $t_{true}^{\left(m\right)}$ and $t_{est}^{\left(m\right)}$ are the *m*th elements of the true data and the estimated value respectively.

#### 4.2.2 Performances of the proposed method

Five different penetration rates (1%, 2%, 3%, 4% and 5%) of floating cars were chosen. For each penetration rate, floating car data were collected from 10 independent simulation runs, and the results are measured with average of RMSE and MAPE.

Section 3 has formulated the missing problem of traffic state in floating car system, especially when floating car penetration rate is low as 1% with missing rate higher than 50%. At such a low penetration rate, for some intervals, traffic state would be missing for all the links. In tensor model, this means the data of a column is completely missing. In this case, it is difficult for conventional methods to effectively estimate the missing values.

**Fig 7:**
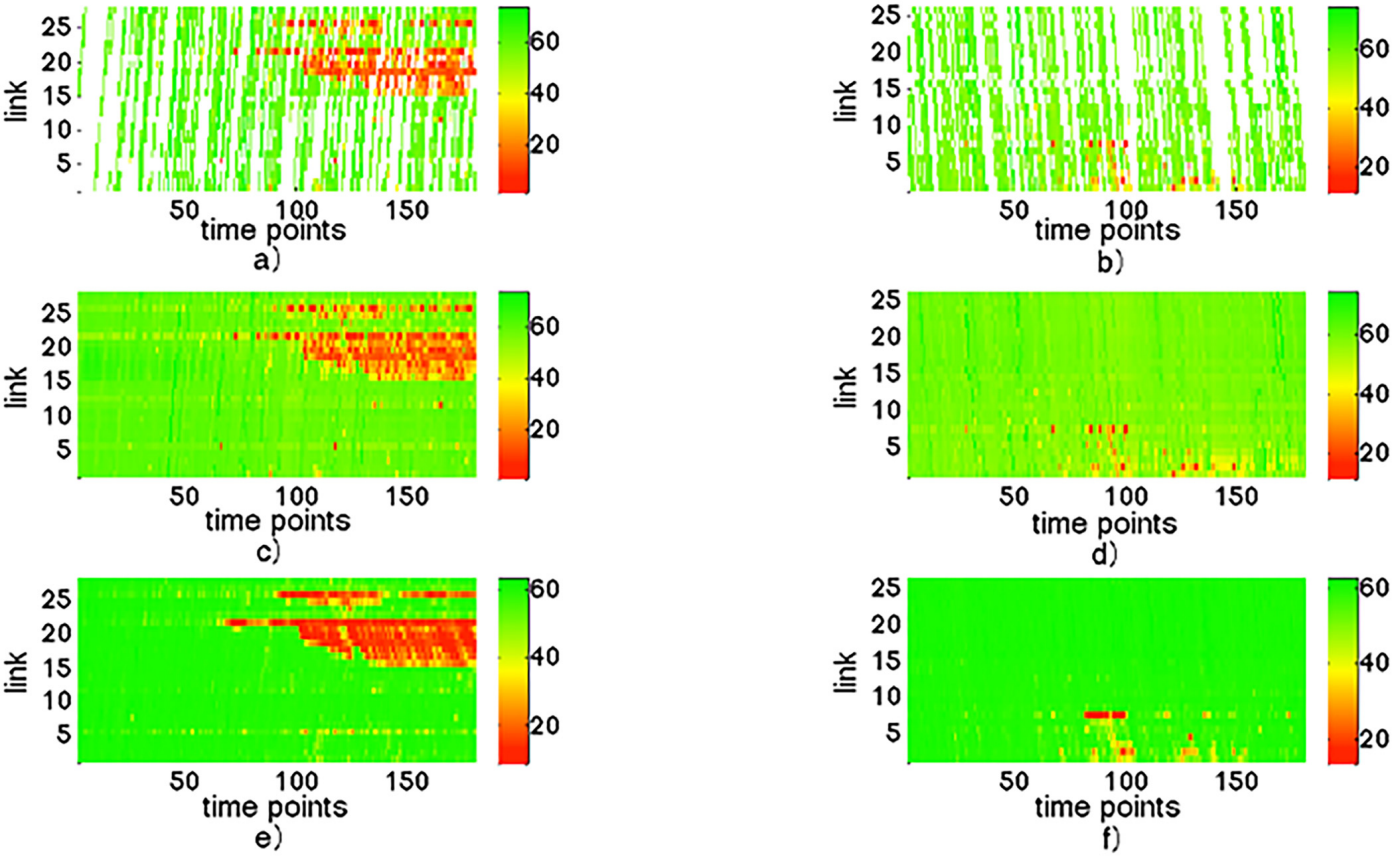
An example of traffic state space-time maps in one day. a) and b): traffic state for direction 1 and direction 2 collected from 1% floating cars. c) and d): traffic state estimation results by proposed method. e) and f): the true traffic state calculated from all vehicles passed through the road networks.

**Fig 8:**
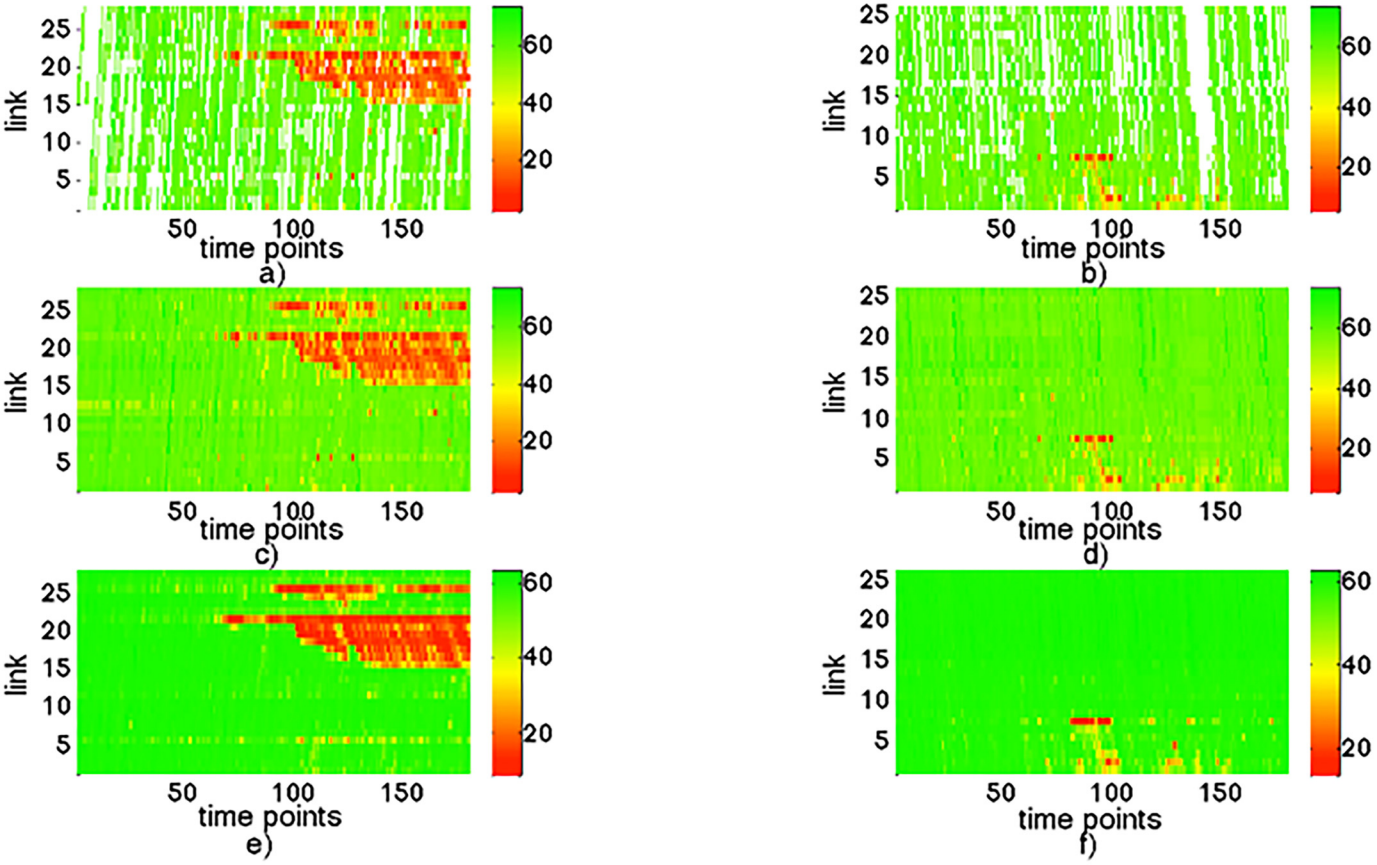
An example of traffic state space-time maps in one day. a) and b): traffic state for direction 1 and direction 2 collected from 2% floating cars. c) and d): traffic state estimation results by proposed method. e) and f): the true traffic state calculated from all vehicles passed through the road networks.

Figs 7 and 8 show a series of daily traffic state maps. The vertical axis represents link and the horizontal axis represents 180 intervals. The heat of red indicates the relative congestion rate, otherwise, green means free flow speed. The top row of Figs 7 and 8 gives the traffic state when floating car penetration rate is 1% and 2%, and it can be seen that the floating car system suffers from a severe missing data problem in these situations. The middle row of Figs 7 and 8 shows the complete traffic state estimated from 1% and 2% penetration rate by using TDI. The bottom row of Figs 7 and 8 is the true traffic state obtained from all vehicles in the simulation. Through Figs 7 and 8, we can observe that the estimation result has a relatively good similarity with true traffic state, which means the proposed method has a satisfying performance. Table 3 gives a quantitative result of traffic state estimation in terms of RMSE and MAPE in 1% and 2% penetration rates.

**Table 3:** The MAPE and RMSE in 1% and 2% penetration.

Penetration rate	1%	2%
**MAPE**	12.51%	12.27%
**RMSE**	9.33km/h	9.18km/h

**Fig 9:**
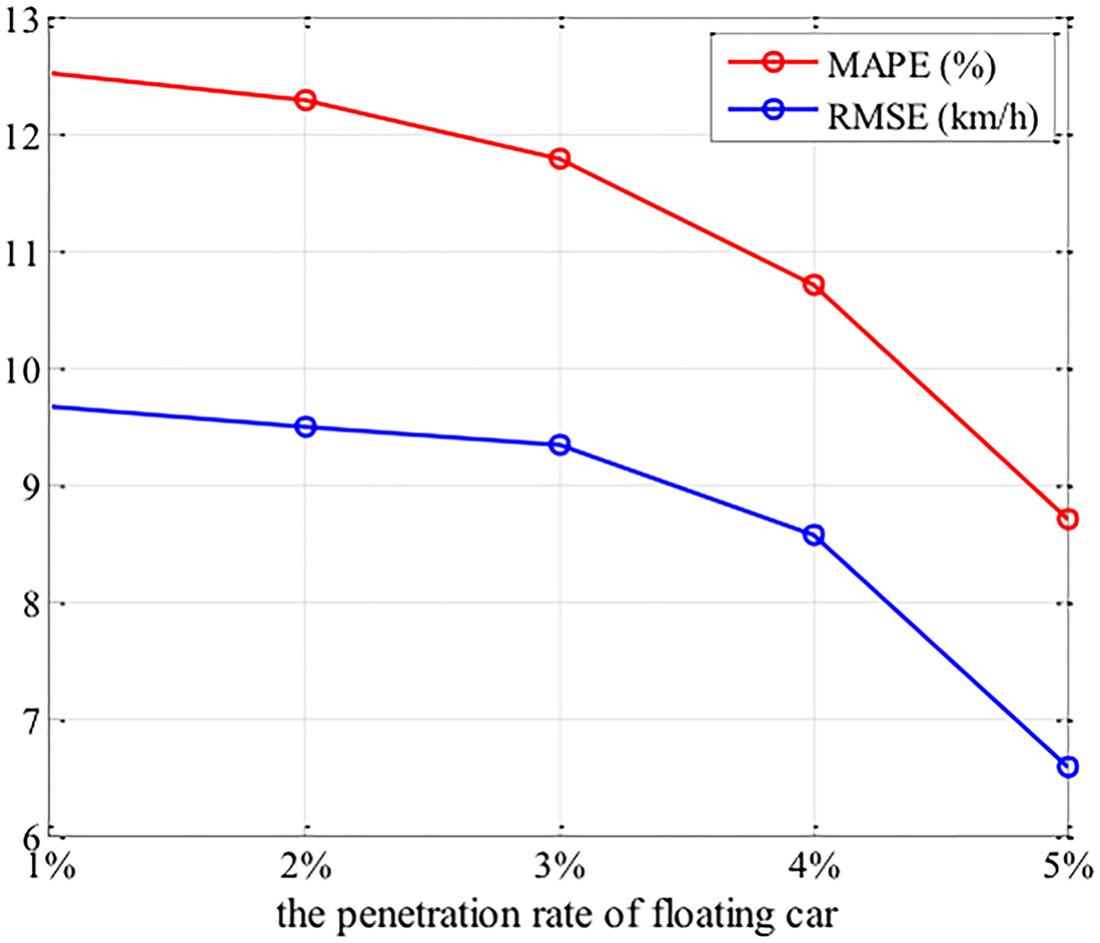
Results of traffic state estimation.

The performances of the proposed method on various penetration rates are shown in Fig 9. From Fig 9, it can be found that the proposed method achieves satisfying results from 1% to 5% penetration rate. The performance of estimation increases with the penetration rate. The RMSE in 5% penetration rate is 7.36km/h, and decreases to only 9.33km/h in 1% penetration, which shows that the performance is still reliable even if the penetration rate is very low.

#### 4.2.3 Comparison with matrix-based methods

The proposed method is compared with matrix-based methods: BPCA and PPCA. The experiments are also performed on the Intel Core 2 Duo and 2GB RAM computer with the same thresholds. Table 4 gives the RMSE of traffic state estimation for the three methods. From 2% to 5% penetration rate, the three methods achieve close performance on traffic state estimation, and TDI just performs slightly better. However, at 1% penetration rate, TDI can still provide acceptable results but the other two methods cannot work. The reason is that both BPCA and PPCA rely only on the correlation of two modes. At such a low penetration rate, one of mode data may be completely missing, i.e., some columns are completely missing in the matrix model. Consequently, BPCA and PPCA will not work in this situation. While TDI is a tensor-based method that can utilize the information of multi-modes simultaneously, hence it can still work well when the penetration rate is very low. In terms of running speed, BPCA is extremely slow, taking over ten minutes. PPCA runs fast and takes less than 1 second. TDI takes around 10s to 20s, slower than PPCA but significantly run faster than BPCA.

**Table 4:** RMSE of traffic state estimation by using TDI, BPCA and PPCA.

Penetration	1%	2%	3%	4%	5%
**TDI**	**9.67km/h**	**9.49 km/h**	**9.33 km/h**	**8.56 km/h**	**6.59 km/h**
**BPCA**	Can’t work	9.73 km/h	9.54 km/h	8.58 km/h	6.60 km/h
**PPCA**	Can’t work	9.79 km/h	9.54 km/h	8.58 km/h	**6.59 km/h**

Table 4 shows that the proposed method outperforms matrix-based methods (BPCA and PPCA) at almost all situations. In low penetration rate, BPCA and PPCA do not work, while TDI can still provide reliable results.

#### 4.2.4 Discussion

According to the above results, the proposed method has satisfying performance in achieving traffic state estimation. Employing a tensor to model traffic state indeed encodes its native structure and multiple modes information simultaneously. Adopting tensor completion method to impute missing traffic state authentically mines the multi-mode similarities of traffic state and estimates missing values effectively. In our study, due to the limitation of simulation, we only considered correlation of link mode, day mode, and interval mode of traffic state. But traffic state has strong correlation in other modes (e.g., week mode) as well, considering these modes and establishing a four-dimensional tensor model may achieve more accurate results.

## 5 Conclusion

This paper proposes a tensor completion method for traffic state estimating from the sparse floating car data. The spatial-temporal traffic state derived from the sparse floating car is utilized to construct the given entries of a traffic state tensor model. Then, the unknown states are estimated by the tensor completion method. The tensor completion method can represent the traffic spatial-temporal information and encode the multi-mode correlations of the traffic state to estimate the unknown states from the low penetration rate of floating cars.

To evaluate the performances of the proposed method, a segment of Interstate Highway I-894 near Milwaukee, Wisconsin was chosen and simulated by VISSIM. Experiments proved the priority of our proposed method: it can estimate the traffic state of the entire network from the sparse floating cars with satisfying accuracy even when the floating car penetration rate is as low as 1%. Compared with matrix-based methods, the proposed method can more effectively dealing with the extreme case in which the floating car is very sparse (e.g., penetration rate is 1%) while the matrix-based methods can not work.

In this study, an assumption is implicitly made in the proposed method: the values on observed entries of the traffic state tensor are reliable. Nevertheless, due to the low penetration of floating cars, only a few cars pass through a link in a certain time interval, and they may not reflect the true traffic state. In this case, the missing entries of the traffic state tensor are estimated from the unreliable entries, and hence may degrade the performance of the proposed method. A future direction is to incorporate the reliability of floating car system into our framework.
